# Multimodal imaging in choroidal osteomas

**DOI:** 10.3205/oc000194

**Published:** 2022-02-25

**Authors:** Sugandha Goel, Sudipta Das, A. Joash Rijey, Debmalya Das

**Affiliations:** 1 Aditya Birla Sankara Nethralaya, Kolkata, West Bengal, India

**Keywords:** choroidal osteoma, multimodal imaging, multicolor imaging, infrared reflectance, osteoma

## Abstract

Choroidal osteoma is a rare benign tumor which is found in the posterior pole of the eye. We herein describe multimodal imaging in two cases of choroidal osteoma. Fundus of our first case showed a yellowish-orange colored subretinal lesion at the posterior pole. Multicolor imaging highlighted the lesion with greenish hue. Infrared reflectance showed hyporeflectance. A dense echogenic plaque persisting in lower gain was noted on B-scan. FFA showed hyperfluorescence with corresponding hypocyanescence on ICG. EDI OCT showed an increase in choroidal thickness with elevated retinal pigment epithelium. The second case showed choroidal osteoma with active choroidal neovascular membrane that responded to intravitreal injection of Ranibizumab.

## Introduction

Choroidal osteoma (CO) is a rare benign tumor of the choroid which is composed of mature bone (trabecular and/or compact) and vascular channels [1]. It was first described by Gass et al. in 1978 [2]. It is mostly unilateral and usually affects healthy young women [3]. It is found in the posterior pole of the eye, juxtapapillary, or in the macular region. We herein report multimodal imaging in two cases of CO.

## Case descriptions

### Case 1

**Figure 1:**
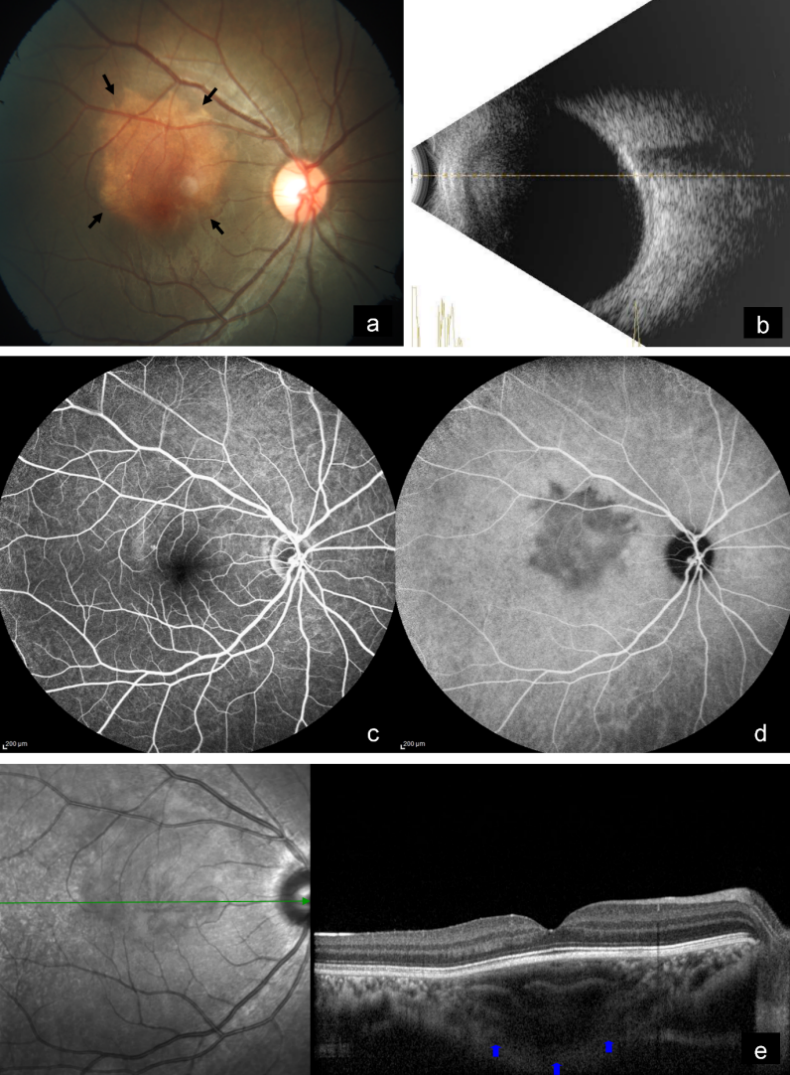
a) Color fundus photograph of the right eye shows a subretinal, yellowish-orange colored lesion in the macula (arrows). b) B-scan shows a densely hyperechoic concave band at the level of the choroid with back shadowing. c) On FFA, the lesion shows fading of hyperfluorescence in the late phase. d) ICG shows hypocyanescence in the corresponding region. e) EDI-OCT shows an increase in choroidal thickness with elevated retinal pigment epithelium (arrows).

**Figure 2:**
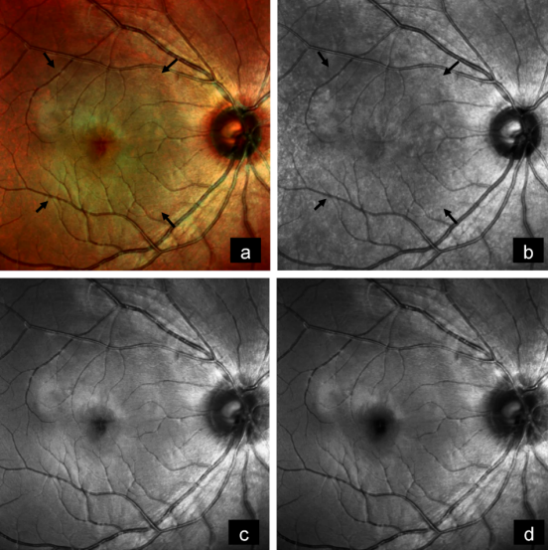
a) Multicolor image highlights the lesion showing greenish hue (arrows). b) Infrared reflectance shows hyporeflectance corresponding to lesion (arrows). c) Lesion cannot be picked up on green reflectance and d) on blue reflectance.

A 14-year-old female came with diminution of vision in the right eye since 2 years. Best-corrected visual acuity (BCVA) was 6/9 and 6/6 in the right and left eye, respectively. Anterior segment examination of both eyes was unremarkable. Fundus examination of the right eye showed a subretinal, yellowish-orange colored lesion of approximately three disc diameters in size in the macula, whereas that of the left eye was normal (Figure 1a (Fig. 1)). B-scan of the right eye demonstrated a densely hyperechoic concave band at the level of the choroid, with back shadowing that persisted on decreased gain (Figure 1b (Fig. 1)). On fundus fluorescein angiography (FFA), the lesion showed granular hyperfluorescence in mid-phase that faded in the late phase (Figure 1c (Fig. 1)). Indocyanine green angiography (ICG) showed hypocyanescence in the corresponding region (Figure 1d (Fig. 1)). Enhanced depth imaging optical coherence tomography (EDI-OCT) showed an increase in choroidal thickness with elevated retinal pigment epithelium (RPE) (Figure 1e (Fig. 1)). Choroidal neovascular membrane (CNV) was not present. Multicolor imaging (MC) highlighted the lesion showing greenish hue (Figure 2a (Fig. 2)). Infrared reflectance (IR) (Figure 2b (Fig. 2)) showed hyporeflectance corresponding to the lesion. The lesion could not be picked up on green reflectance (GR) (Figure 2c (Fig. 2)) and blue reflectance (BR) (Figure 2d (Fig. 2)). A diagnosis of CO was made, and the patient was asked to come for regular follow-up.

### Case 2

**Figure 3:**
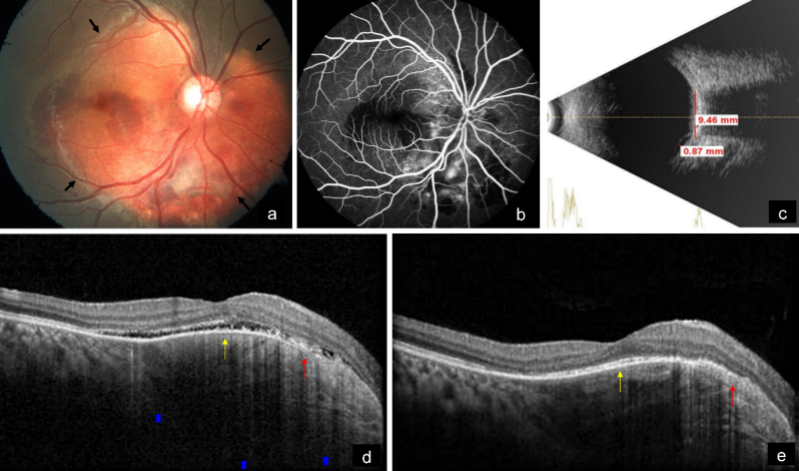
a) Color fundus photograph of the right eye shows a subretinal, yellowish-orange colored lesion in the peripapillary area involving the macula (arrows) with subretinal hemorrhage over the macula and inferior to the optic disc. b) On FFA, the lesion shows granular hyperfluorescence with areas of blocked fluorescence in mid-phase. c) B-scan shows a hyperechoic choroidal lesion with back shadowing. d) EDI-OCT shows an increase in choroidal thickness (blue arrows) with elevated RPE, along with CNV (red arrow) and subretinal fluid (yellow arrow). e) EDI-OCT shows scarred CNV (red arrow) with resolution of subretinal fluid (yellow arrow) after 1 month of intravitreal injection of Ranibizumab.

**Figure 4:**
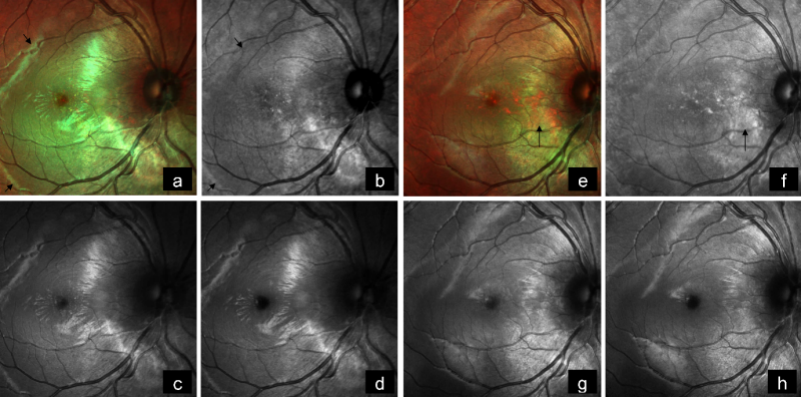
a) Multicolor image highlights the lesion showing greenish hue (arrows). b) Infrared reflectance shows a dark area corresponding to the tumor (arrows). c) The lesion could not be picked up on green reflectance, and d) on blue reflectance. e) On follow-up, multicolor image shows orange discoloration at the posterior pole, suggestive of RPE atrophy overlying the CO (arrow). f) Infrared reflectance shows white areas corresponding to RPE atrophy (arrow). g) Changes are not evident in green reflectance, and h) in blue reflectance.

A 19-year-old female came with diminution of vision in the right eye since 2 weeks. BCVA was 6/18 and 6/6 in the right and left eye, respectively. Anterior segment examination of both eyes was unremarkable. Fundus examination of the right eye showed a subretinal, yellowish-orange colored lesion of approximately five disc diameters in size in the peripapillary area involving the macula with areas of subretinal hemorrhage over the macula and inferior to the optic disc, whereas that of the left eye was normal (Figure 3a (Fig. 3)). On FFA, the lesion showed granular hyperfluorescence with areas of blocked fluorescence in mid-phase (Figure 3b (Fig. 3)). B-scan demonstrated hyperechoic choroidal lesion with back shadowing (Figure 3c (Fig. 3)). EDI-OCT showed an increase in choroidal thickness with elevated RPE, along with CNV and subretinal fluid (Figure 3d (Fig. 3)). MC highlighted the lesion, showing greenish hue (Figure 4a (Fig. 4)). IR (Figure 4b (Fig. 4)) showed a dark area in the corresponding region. The lesion could not be picked up on GR (Figure 4c (Fig. 4)) and BR (Figure 4d (Fig. 4)). A diagnosis of CO with active CNV was made. The patient was subjected to intravitreal injection of Ranibizumab (Accentrix). One month post injection, her BCVA improved to 6/9 in the right eye. MC showed orange discoloration at the posterior pole, suggestive of RPE atrophy overlying the CO (Figure 4e (Fig. 4)). IR (Figure 4f (Fig. 4)) showed white areas corresponding to RPE atrophy. Changes could not be appreciated in GR (Figure 4g (Fig. 4)) and BR (Figure 4h (Fig. 4)). EDI-OCT showed scarred CNV with resolution of subretinal fluid (Figure 3e (Fig. 3)).

## Discussion

CO is an ossifying tumor involving the choroid. Its natural course may include tumor growth, calcifcation and decalcifcation; vision depends on development of CNV and retinal changes due to decalcification [4]. Though present from birth, it is usually diagnosed during the second or third decade of life. Though its etiology is unclear, it is usually diagnosed due to its typical clinical features of yellowish-orange colored subretinal lesion at the posterior pole and a dense echogenic plaque persisting even in lower gain on B-scan. FFA shows granular hyperfluorescence of the large yellow patches that fades on late films, or they may exhibit late staining. EDI-OCT has been able to reveal the presence of bone lamella, tubular lamella with optically empty center, vascular channels, and trabecular bone in patients with CO. MC helps in highlighting the lesion clearly. Venkatesh et al. have reported the role of MC in CO [5]. In their study, MC images showed color variations depending upon the reflectivity of the tumor material and associated RPE atrophy. Green color was noted in calcified CO tumor, while decalcified CO tumor showed no color change. RPE atrophy were seen as bright orange areas. Green and blue reflectance images were not able to pick up the CO lesion. IR images showed calcified CO lesions as hyporeflectance (dark) areas, while decalcified lesions were seen as iso reflectance (normal) areas. Overlying RPE atrophy on IR were seen as white areas [5]. In both of our cases, green color on MC and hyporeflectance on IR suggest calcified CO tumor. Thus, change in reflectance of the IR and MC images can be used as an indicator to assess the extent of tumor decalcification. CO must be differentiated mainly from amelanotic choroidal melanoma, choroidal nevus, choroidal hemangioma, choroidal metastasis, organized subretinal hemorrhage, sclerochoroidal calcification, posterior scleritis, and age-related macular degeneration. However, its distinct clinical and ultrasonographic presentation aids in the diagnosis. Most patients do not require treatment. CNV should be ruled out on long-term follow-up [6]. In the presence of CNV, treatment can be given with anti-VEGF similar to our second case [7], [8]. Transpupillary thermotherapy [9] and photodyamic therapy (PDT) or their combined treatment [10] have also been shown to be effective in cases of CNV.

## Notes

### Competing interests

The authors declare that they have no competing interests.
